# Detecting selection in low-coverage high-throughput sequencing data using principal component analysis

**DOI:** 10.1186/s12859-021-04375-2

**Published:** 2021-09-29

**Authors:** Jonas Meisner, Anders Albrechtsen, Kristian Hanghøj

**Affiliations:** Department of Biology, The Bioinformatics Centre, University of Copenhagen, Copenhagen, Denmark

## Abstract

**Background:**

Identification of selection signatures between populations is often an important part of a population genetic study. Leveraging high-throughput DNA sequencing larger sample sizes of populations with similar ancestries has become increasingly common. This has led to the need of methods capable of identifying signals of selection in populations with a continuous cline of genetic differentiation. Individuals from continuous populations are inherently challenging to group into meaningful units which is why existing methods rely on principal components analysis for inference of the selection signals. These existing methods require called genotypes as input which is problematic for studies based on low-coverage sequencing data.

**Materials and methods:**

We have extended two principal component analysis based selection statistics to genotype likelihood data and applied them to low-coverage sequencing data from the 1000 Genomes Project for populations with European and East Asian ancestry to detect signals of selection in samples with continuous population structure.

**Results:**

Here, we present two selections statistics which we have implemented in the PCAngsd framework. These methods account for genotype uncertainty, opening for the opportunity to conduct selection scans in continuous populations from low and/or variable coverage sequencing data. To illustrate their use, we applied the methods to low-coverage sequencing data from human populations of East Asian and European ancestries and show that the implemented selection statistics can control the false positive rate and that they identify the same signatures of selection from low-coverage sequencing data as state-of-the-art software using high quality called genotypes.

**Conclusion:**

We show that selection scans of low-coverage sequencing data of populations with similar ancestry perform on par with that obtained from high quality genotype data. Moreover, we demonstrate that PCAngsd outperform selection statistics obtained from called genotypes from low-coverage sequencing data without the need for ad-hoc filtering.

**Supplementary Information:**

The online version contains supplementary material available at 10.1186/s12859-021-04375-2.

## Introduction

Natural selection is the main driver of local adaptation. Instead of tracing the adaptive phenotypic trait, a “reverse ecology” approach is commonly applied [13], where the genetic variant encoding for a beneficial trait is first identified followed by the underlying mechanism of the adaptive phenotype. This enables mapping of the genetic architecture of phenotypic adaptability driven by natural selection ([6] for review on human populations). A common approach to identify candidates under selection is based on outliers in an empirical distribution of differentiation between two or more groups of predefined populations. In it simplest form, it finds the variants with the biggest difference in allele frequency between two predefined populations. One of many methods based on this notion is Population Branch Statistics [31], an estimator of genetic differentiation based on allelic changes estimated with the fixation index $(F_{ST})$. It identifies candidate regions as strong deviations from an empirical distribution between a target population, a closely related sister population and an outgroup. However, homogeneous discrete groupings of the populations is required for many of these models, albeit exceptions exist [2].

The reduced expenses for whole genome DNA sequencing, thanks to advanced High-throughput DNA sequencing technologies, has facilitated larger sample sizes in population genetics studies in the recent years, including samples with similar genetic ancestry [4, 14, 20, 26, 29, 30]. Identifying signatures of selection in populations of similar genetic ancestry can results in arbitrary population assignments when using methodologies that require discrete groups of populations. This can lead to reduced power and increased false positive rates as allele frequencies are estimated from non-homogeneous populations. Instead of coercing samples into groups, an alternative approach is to account for the continuous cline of genetic differentiation in the selection analysis. Recent studies has shown that principal components analysis (PCA) of genetic data can detect signals of selection in continuous populations [7, 15]. Briefly, the idea is to use PCA to infer a weight for each variant which is scaled to reflect genetic drift. Variants with deviating statistics from the null distribution of what is expected under pure drift are candidates for selection. This approach has been applied to several dataset, including populations of humans [3, 7, 14], wheat [23], cod [27], turbots [19], and tiger mosquito [10].

Two commonly used software that accounts for continuous population differentiation when performing selection scans are FastPCA [7] and pcadapt [15, 24]. Both software use called genotypes as input to obtain the top *K* principal components (PCs) and variant weights through a truncated singular value decomposition (SVD) [11, 25]. However, they differ in their derived test statistics. pcadapt uses robust Mahalanobis distance [16] to evaluate all top *K* PCs for estimating *z*-scores, whereas FastPCA test normalized variant weights for each PC separately. Both test statistics follow $\chi ^2$ distributions from which a *p* value for each polymorphic site is obtained.

In this study, we extended the FastPCA [7] and pcadapt [15] selection statistics to account for genotype uncertainty by leveraging the PCs and variant weights estimated iteratively in the PCAngsd framework [18] using genotype likelihoods. This allows us to analyze low-coverage data and naturally impute missing data based on individual allele frequencies estimated from the top *K* inferred PCs. We apply the novel methods to populations of East Asian ancestry and European ancestry using the low-coverage data of the 1000 Genome Project [4] and demonstrate that we can identify known signatures of selection within these two ancestries. The candidates under selection were verified using the corresponding high quality genotype data from the 1000 Genome Project. The test statistics are implemented in the PCAngsd framework [18] that is available at https://github.com/rosemeis/pcangsd.

## Materials and methods

We assume that variable sites are diallelic and the major and minor allele are known such that genotypes are expected to follow a Binomial model. In low-coverage sequencing data, genotypes are unobserved and genotype likelihoods are therefore used instead to account for the uncertainty in sequencing process. We use the iterative procedure in PCAngsd [18] to estimate individual allele frequencies that can be seen as the underlying parameters in the Binomial sampling processes of the genotypes accounting for population structure. In the following, we will denote *N* as the number of individuals and *M* as the number of sites. We can then define the posterior genotype dosage as follows for individual *i* in site *j*

1 $$\begin{aligned} {\mathbb {E}}[G_{ij} \,|\, X_{ij}, {\hat{\pi }}_{ij}] = \sum ^2_{g=0} g\, P(G_{ij} = g \,|\, X_{ij}, {\hat{\pi }}_{ij}), \end{aligned}$$

for $i=1, \dots , N$ and $j=1, \dots , M$, where $P(G_{ij} = g \,|\, X_{ij}, {\hat{\pi }}_{ij})$ is the posterior genotype probability of genotype *g* with *X* being the observed sequencing data, and ${\hat{\pi }}$ being the individual allele frequency. Details of deriving the posterior genotype from genotype likelihoods can be found in the Additional file 1 (Equation S1-S2). Missing data is imputed based on population structure based on the posterior genotype dosages. We standardize the dosage under the assumption of a Binomial model,

2 $$\begin{aligned} y_{ij} = \frac{ {\mathbb {E}}[G_{ij} \,|\, X_{ij}, {\hat{\pi }}_{ij}] - 2{\hat{f}}_j}{\sqrt{2{\hat{f}}_j (1 - {\hat{f}}_j)}}. \end{aligned}$$

Here ${\hat{f}}$ is the estimated allele frequency at site *j* based on all of the samples. We then perform truncated SVD [11] on the full standardized data matrix ($N \times M$) to extract the top *K* principal components (PCs) that capture population structure in the dataset

3 $$\begin{aligned} \hat{\mathbf {Y}} = \mathbf {U}_{[1:K]} \mathbf {S}_{[1:K]} \mathbf {V}_{[1:K]}^T, \end{aligned}$$

where $\mathbf {U}_{[1:K]}$ represents the captured population structure of the individuals and $\mathbf {V}_{[1:K]}$ represents the scaled variant weights, while $\mathbf {S}_{[1:K]}$ is the diagonal matrix of singular values. This low-rank approximation along with the standardized matrix $\mathbf {Y}$ are all we need to estimate the two test statistics for low-coverage sequencing data.

### FastPCA statistic

The selection statistic derived in Galinsky et al. [7], hereafter referred to as FastPCA, tries to detect selection by looking for variants that significantly differentiate from genetic drift along an axis of genetic variation. They define the selection statistics for the *k*-th principal component to be the properly normalized variant weights, using the properties of an eigenvector, such that they are standard normal distributed. The selection statistics are then defined as follows in our setting for genotype likelihood data

4 $$\begin{aligned}d_{jk} = v_{jk} \sqrt{M}, \end{aligned}$$

5 $$\begin{aligned}&d_{jk} \sim {\mathcal {N}}(0,1), \end{aligned}$$

6 $$\begin{aligned}&d_{jk}^{2} \sim \chi ^2_{1}, \end{aligned}$$

for $j=1, \ldots , M$ and $k=1, \ldots , K$. $v_{jk}$ is the variant weight for the *k*th component at site *j*. The squared statistic will then follow a $\chi ^2$-distribution with 1 degree of freedom. This statistic is implemented in the PCAngsd framework and referred to as PCAngsd-S1.

### pcadapt statistic

The test statistic implemented in pcadapt [15] is based on a robust Mahalanobis distance of the standardized estimates in a multiple linear regression for each site. The regression model is defined as follows in our setting for genotype likelihood data

7 $$\begin{aligned} \mathbf {y}_j = \mathbf {U}_{[1:K]} \varvec{\beta }_j + \varvec{\epsilon }_j, \end{aligned}$$

for $j=1, \dots , M$, with $\varvec{\beta }_j$ being the regression coefficients, and $\varvec{\epsilon }_j$, the residual vector for site *j*. The coefficients are easily derived using the normal equation and properties of the previously computed truncated SVD (Eq. 3), thus $\varvec{\beta }_j = \mathbf {S}_{[1:K]} \mathbf {V}_{[j,1:K]}$. A *z*-score of the regression coefficients in site *j* are defined as

8 $$\begin{aligned} \mathbf {z}_j =\varvec{\beta }_j/\sqrt{\frac{(\mathbf {y}_j - \hat{\mathbf {y}}_{j})^T (\mathbf {y}_j - \hat{\mathbf {y}}_{j})}{N-K}}, \end{aligned}$$

with $\hat{\mathbf {y}}_{j}$ being the vector of low-rank approximations in site *j* (Eq. 3). The test statistic is computed as a robust Mahalanobis distance of $\mathbf {z}_j$, where the squared distance will be $\chi ^{2}_K$ distributed as described in Luu et al. [15]. We use standardized expected genotypes $y_{ij}$ (Eq. 2) for genotype likelihood data, hereafter referred to as PCAngsd-S2, instead of using known genotypes as pcadapt. Note, that we correct for inflation using the genomic inflation factor [5], inline with the recommendations [15], in all analysis based on the pcadapt or PCAngsd-S2 statistics. See QQ-plot in Additional file 1: Figure S2 and S3 for examples of the uncorrected PCAngsd-S2 test statistic.

### 1000 genomes project data

We used data from the 1000 Genomes Project (phase3) [4] to test the two selection statistics implemented in the PCAngsd framework. Specifically, we tested two sets of populations, one with East Asian ancestry with 400 unrelated individuals from four East Asian populations, Han Chinese in Beijing (CHB), Han Chinese South (CHS), Chinese Dai in Xishuanagbanna (CDX), Kinh in Ho Chi Minh City (KHV), and one with European ancestry with 404 unrelated individuals from four European populations, Utah residents with Northern and Western European Ancestry (CEU), British in England and Scotland (GBR), Iberian populations in Spain (IBS), Toscani in Italy (TSI).

For all the selected individuals, we have both high quality genotype (HQG) data and low-coverage sequencing data available from the 1000 Genomes Project (phase3) (see [4] for details on the HQG data). The low coverage data is whole genome sequencing data with a mean depth of coverage around 6X (Additional file 1: Figure S1).

#### Analyses on polymorphic sites from the high quality genotype data

To directly compare PCAngsd against pcadapt and FastPCA, where the latter two only takes called genotypes as input, we restricted the selection analyses to polymorphic sites with a minimum allele frequency of 5% in the HGQ data. In total 5.8 and 6 million polymorphic sites are retained in the Asian and European population sets, respectively.

Restricting to the polymorphic sites in HQG data, we calculated genotype likelihoods (GL) from the low-coverage data using ANGSD with minimum mapping quality of 20 and minimum base quality of 30 [9]. We used the GL data as input to PCAngsd to compute the two selection statistics (PCAngsd-S1, PCAngsd-S2) for the population sets. To verify the results obtained from the low-coverage data, we also analyzed the same individuals in the HQG data using PCAngsd, pcadapt (default settings), and FastPCA (fastmode:YES, following [7]).

We also tested the performance of pcadapt and FastPCA on low-coverage data by calling genotypes using bcftools [12] with minimum mapping quality of 30, minimum base quality of 20, and disabling BAQ (–no-BAQ) to resemble the filters used with ANGSD. We restricted the analyses to the polymorphic sites in the HQG described above. From the called genotypes from low-coverage sequencing data, we generated two datasets: One excluding all genotype calls with genotype quality $<20$ (hereafter referred to as CG standard) and one including all called genotypes (hereafter referred to as CG*).

#### Analyses on low coverage data

We also used the low-coverage sequencing data to test the performance of PCAngsd without prior knowledge on polymorphic sites. We used ANGSD with the same mapping (30) and base quality (20) filters described above to calculate GL. Variable sites were identified using a likelihood ratio test (-SNP_pval $10^{-6}$) and a minimum allele frequency filter on 0.05 (-minmaf 0.05). To remove false positive variable sites, we next applied a callability filter that excludes genomic regions of low quality and complexity. The filter is based on the sequencing depth and mapping quality across samples, thus, no external information is required. In total, we identified 4.1 million and 4.6 million polymorphic sites in the Asian and European population sets, respectively.

#### Read length bias in low coverage sequencing data

The low coverage sequencing data from the 1000 Genomes Project consists of multiple difference sequencing sources with highly variable sequencing length. Variable sequencing length can introduce a bias in population genetics analyses, particularly for low structure analyses. In the PCA of the East Asian samples based on polymorphic sites identified from the low coverage sequencing data, PC2 correlates with the sequencing length of the samples (Additional file 1: Figure S7). To identify genomic sites where the GLs correlate with the sequencing length, we conducted a logistic regression analyses tailored for low coverage sequencing data using ANGSD (-doAsso 5, [8]), where the trait/phenotype is the samples stratified the into two groups based on their read length ($<99$bp and $\ge 99$bp) and their sample population of origin was used as covariates to ensure we did not identify genomic sites driven by population differentiation. We removed sites with a p-value $< 10^{-3}$.

## Results and discussion

To test the performance of the two selection statistics (PCAngsd-S1 and PCAngsd-S2), implemented in PCAngsd, on continuous genetic differentiation in low-coverage data sets, we used data from the 1000 Genomes Project [4]. We tested four populations with East Asian ancestry and four populations with European ancestry and identified known signatures of selection in both ancestries. We compared the results to FastPCA and pcadapt applied to HQG data and two data sets based on called genotypes from the low-coverage data, CG standard where all genotype calls with a genotype quality lower than 20 were excluded and CG* containing all called genotypes.

**Fig. 1:**
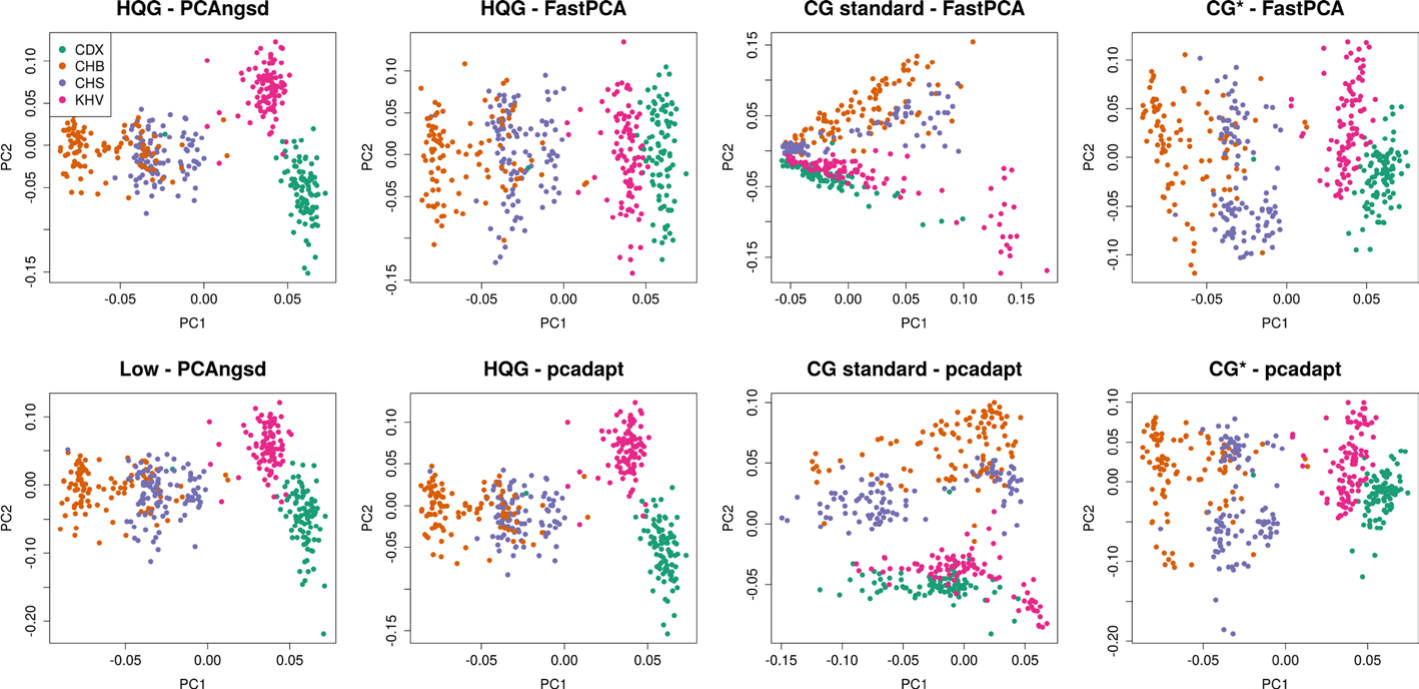
PCA plots of the samples from the four East Asian populations using PCAngsd, FastPCA and pcadapt HQG: High quality genotype data, Low: Low-coverage data, CG standard: Called genotypes from low-coverage data with genotype quality threshold on 20, CG*: Called genotypes from low-coverage data

We applied the selection statistics to 400 individuals from four populations (CHB, CHS, KHV, CDX) with East Asian ancestry. First, we performed PCA on the GL data using PCAngsd where we observed a continuous separation between the northern (CHB, CHS) and southern (KHV, CDX) populations on the first principal component (PC) (Fig. 1). FastPCA and pcadapt obtained a similar pattern using the HQG data (Fig. 1). PC2 obtained from PCAngsd and pcadapt separate the Vietnamese Kinh population (KHV) and Chinese Dai population (CDX) (Fig. 1). When applied to the CG standard data, FastPCA and pcadapt could not recover the continuous separation on PC1. Instead we observe within population variance driven by the bias from genotype calling on low depth data when genotype quality filters are applied [21]. Therefore, CG standard data was not used for downstream selection scan comparisons. The PCA obtained from genotype data without quality filter CG* did not show the same problems and recovered the continuous separation on PC1 and was included in the following selection scan analyses Figure 1.

**Fig. 2:**
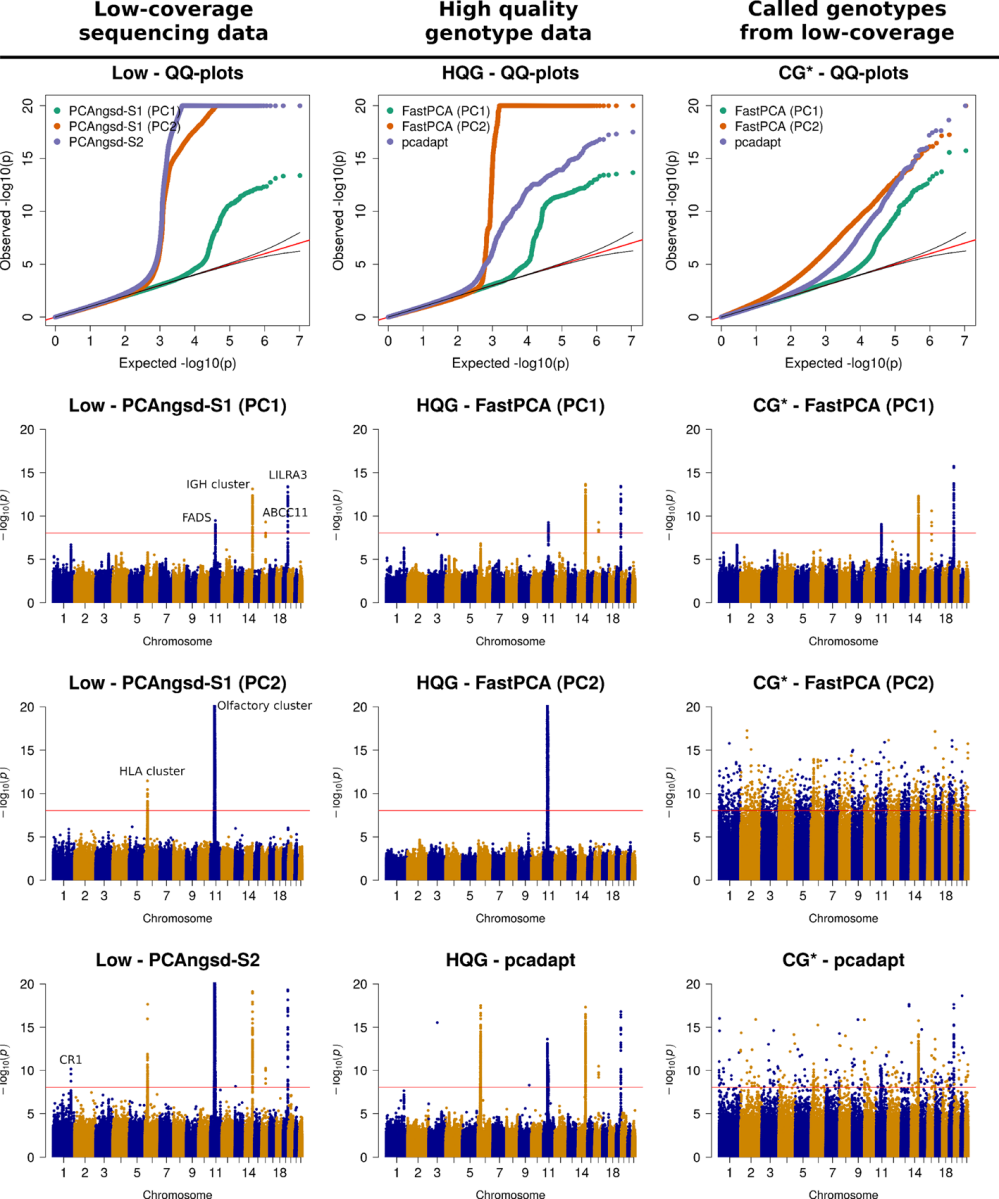
Selection scan of East Asian populations. QQ and Manhattan plots of the selection statistics from PCAngsd, FastPCA and pcadapt applied to the four East Asian populations obtained. Red horizontal line is the Bonferroni adjusted significance level. PCAngsd-S2 and pcadapt has been corrected for genomic inflation. HQG: High quality genotype data, Low: Low-coverage data, CG*: Called genotypes from low-coverage data

We applied the test statistics on the variant weights inferred along the two PCs and scan for genomic regions with significant differentiation on the continuous north-to-south cline on PC1 and separation of KHV and CDX on PC2. We identify several candidates under selection along PC1 (Fig. 2). After multiple testing correction using Bonferroni (*p*-value $<9\times 10^{-9},~\alpha =0.05$), we find significant signals of differentiation in variants overlapping *FADS2* (chr11), *IGH* cluster (chr14), *ABCC11* (chr16), and *LILRA3* (chr19) (see S13A for an example of the effect of selection across the PC1 gradient). These signatures of selection have been described in previous studies of selection on continuous differentiation in Han Chinese populations [3, 14]. Interestingly, PCAngsd also identifies a genomic region overlapping *CR1* on the low coverage data, previously described by Chiang and colleagues [3] and the NIPT data [14]. We find a similar signal using the other software on the HQG although not significant. FastPCA and pcadapt find the same candidates with significant differentiation when applied to the HQG data.

Both PCAngsd and pcadapt identify population structure on PC2 separating CDX and KHV. FastPCA can obtain higher accuracy by increasing the number of power iterations but by default it assigns the number of power iterations to *k*, the number of eigenvectors considered. PCAngsd and pcadapt identify the same two significant candidate regions: *HLA-cluster* (chr6) (also observed in [3]) and *Olfactory cluster* (chr11) (Fig. 2). The variants overlapping the Olfactory cluster show strong LD pattern on both sides of the centromere, a challenging region to assemble potentially resulting in systematic biases, however, we do note that the pattern is present both on the HQG and low-coverage data (Fig. 2 and Additional file 1: Figure S2 ). PCAngsd-S2 and pcadapt identify a single significant variant on chr3 and chr9 in the HQG data. Following a test for Hardy-Weinberg equilibrium (HWE) accounting for population structure [17], we find that these two variants are the only top hits among selection candidates that significantly deviate from HWE (Additional file 1: Table S1). This indicates genotype calling related biases as the variants are not candidates under selection in the low-coverage sequencing data.

When FastPCA and pcadapt are applied to the low depth data, CG*, not all of these signals are identified despite PC1 separating the four populations. We observed highly inflated statistics with significant false positive signals present genome-wide blurring the signals observed on the HQG data (Fig. 2).

**Fig. 3:**
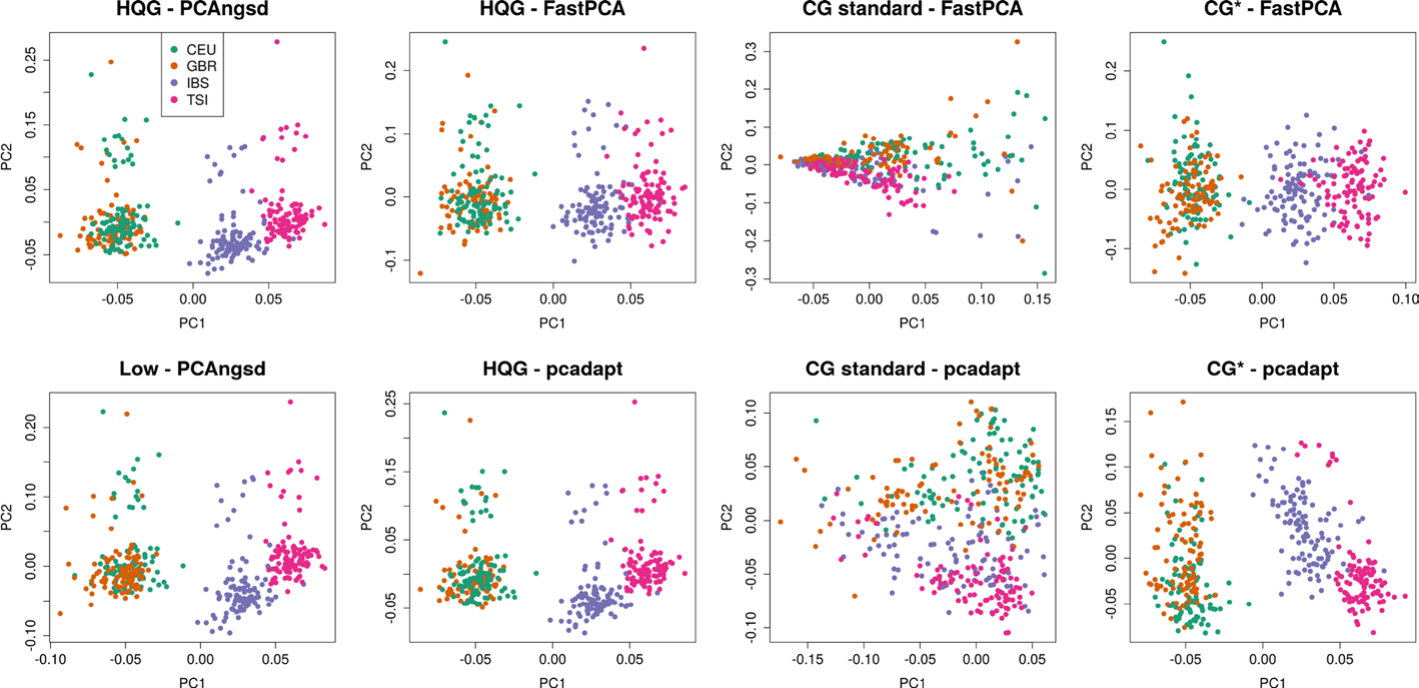
PCA plots of the samples from the four European populations obtained from PCAngsd, FastPCA and pcadapt. HQG: High quality genotype data, Low: Low-coverage data, CG*: Called genotypes from low-coverage data

Similarly to the populations with East Asian ancestry, we also performed selection scans of 404 individuals from four populations (CEU, GBR, IBS, TSI) with European ancestry. We know from previous research that lactase persistence and skin and hair pigmentation distributions show a north-south cline within European populations [1, 22, 28], where the Northern European populations have higher lactase persistence and lighter pigmentation than the Southern European populations. We first performed PCA on the GL data using PCAngsd [18] (Fig. 3) where we observed a continuous separation between the northern (CEU, GBR) and southern (TSI, IBS) populations on the first PC. FastPCA and pcadapt obtained a similar pattern on the HQG data (Fig. 3). As for the East Asian scenario, FastPCA and pcadapt could not recover the continuous separation on PC1 on the CG standard data which was excluded from further analysis. The PCA obtained from CG* data set recovered the continuous separation on PC1 and was used in the following selection scan analyses Figure 3

**Fig. 4:**
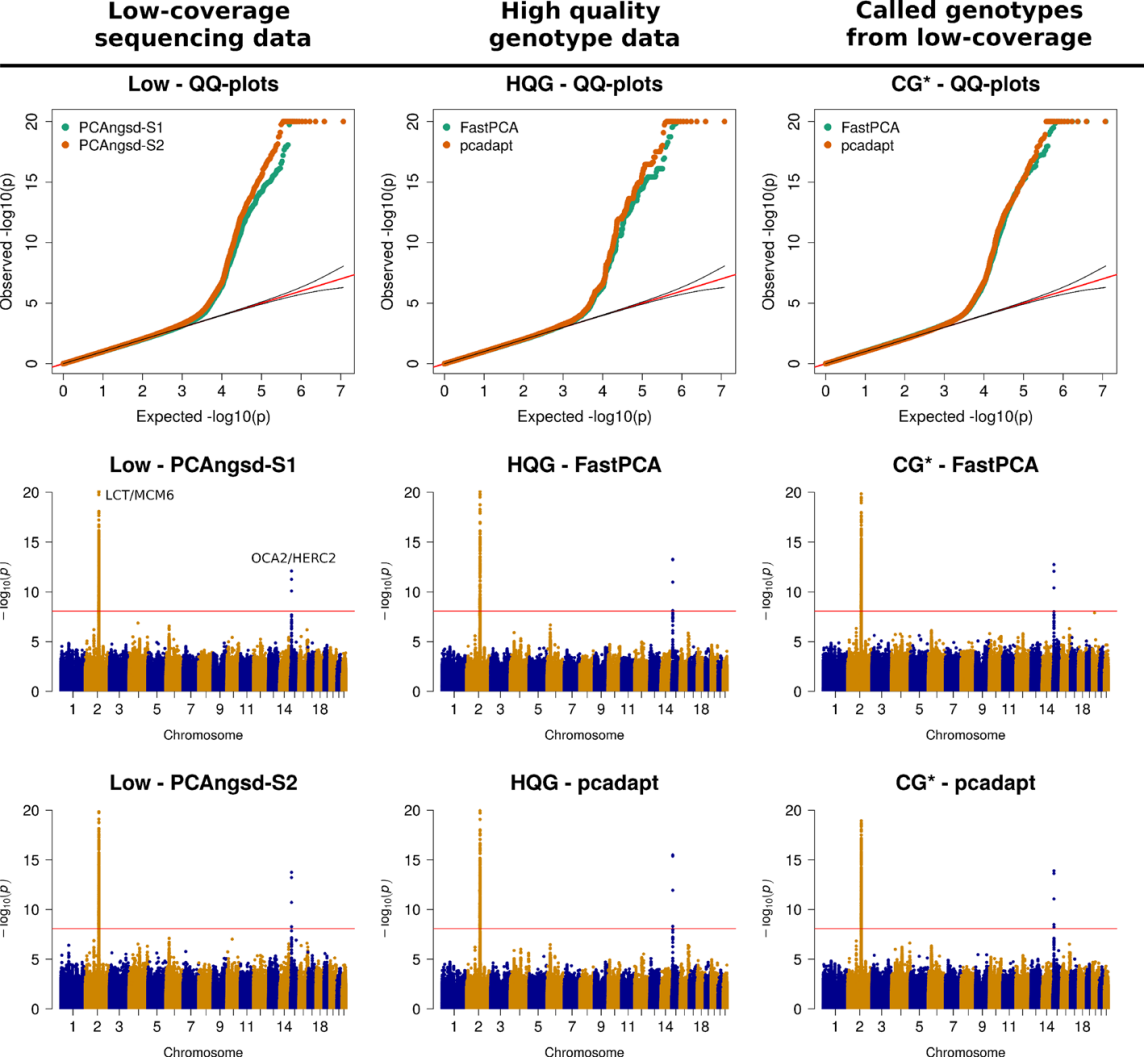
Selection scan of European populations. QQ and manhattan plots of the selection statistics from PCAngsd, FastPCA and pcadapt applied to the four European populations obtained. Red horizontal line is the Bonferroni adjusted significance level. PCAngsd-S2 and pcadapt has been corrected for genomic inflation. HQG: High quality genotype data, LOW: Low-coverage data, CG*: Called genotypes from low-coverage data

Next, we calculated the selection statistics along PC1 that display a north-south cline in the European populations. We find that both PCAngsd-S1 and PCAngsd-S2 statistics behaves as expected under the null hypothesis for most sites(Fig. 4). Similarly the statistics obtained from FastPCA and pcadapt follows the expectation, although, the latter required genomic inflation correction [5], on both HQG and CG*. After multiple testing correction, all software identify two genomic regions with significant genetic differentiation overlapping two gene clusters: *LCT*/*MCM6* (chr2) (see S13B for an example of the effect of selection across the PC1 gradient) and *OCA2*/*HERC2* (chr15) (Fig. 4). These results are inline with previous research on these populations [1, 22, 28].

For genotype calling from low-coverage data uncertain genotype calls are often excluded by applying a genotype quality threshold. After applying a genotype quality threshold of 20 both FastPCA and pcadapt identify within population biases on the first PC (see Figs. 1, 3). However, as the second PC to some extent recover the population structure, we applied FastPCA and pcadapt to the standard genotype calls. In the selection scan of the East Asian populations pcadapt recovered the same candidates regions as the HQG data, whereas FastPCA identified many false positive regions both on PC1 and PC2 (Additional file 1: Figure S4). For the European populations, we observe highly inflated statistics on both PCs and many false positive selection signatures were identified genome-wide by both software (Additional file 1: Figure S5). From these observations, it is evident that genotype calling of low-coverage data requires ad-hoc filters for each test scenario. Similarly, in a recent low-coverage study Chiang and colleagues also used extensive filters, including machine learning algorithms, to exclude outlier samples and variants prior to computing the selection statistics for the Han Chinese population [3]. In contrast, we show that the PCAngsd framework consistently obtain well-behaving selection statistics in both scenarios from low-coverage data without the need for ad-hoc quality filters on either variant calls or sample selection.

To test the accuracy of PCAngsd on low coverage sequencing without restricting to polymorphic sites from the HQG data, we used ANGSD for variant detection. After quality filtering (see “Analyses on low coverage data” section), we obtain similar PCA and selection statistics in both ancestries (Additional file 1: Figure S9, S10) to those obtained from the HQG variant set, although PCAngsd-S2 identify slightly more false positive signals of selection in the data set with European ancestry. These results are robust to downsampling to only half of the sequencing data (mean depth $\sim 3$X) (Additional file 1: Figure S11, S12). We applied a simpler version of the method implemented in PCAngsd to ultra low-coverage sequencing ($<0.1$X) [14].

A limitation of the PC-based selection scans is their capability of detecting selection in scenarios of non-continuous population structure. We show an example of this in Additional file 1: Figure S6, where we have applied the three software to three populations with distinct ancestry (European (CEU), East Asian (CHB), African (YRI)). As also shown in the original study of FastPCA [7], it has low power in data sets with higher $F_{ST}$ between the populations, where we see deflated test statistics due to being inversely scaled with the inferred large eigenvalues of the corresponding tested PC for PCAngsd-S1 and FastPCA. We see the opposite pattern for PCAngsd-S2 and pcadapt, where the test statistics are very inflated, even after correction with genomic control, leading to many false positives. In scenarios with discrete structure other methods are more appropriate such as $F_{ST}$ or population branch statistic based selection scans are more appropriate. However, even if you have discrete population clusters then you can try to use the PC-based method using the individuals within a cluster as illustrated in this paper where we can detect selection within the Europeans or East Asian clusters. The appropriateness of the PC-based selection scan can easily be evaluated from the QQ-plot.

For PC-based selection scan methods it is also important to ensure that the PC’s reflect structure in the data and not e.g. relatedness or technical artifacts. An example of the latter can be found in Additional file 1: Figure S7, where PC2 separates samples from East Asia based on their read length and not population structure. The PCA of European ancestries did not show such bias S8. In method “Read length bias in low coverage sequencing data” section, we show how to exclude sites that drive this particular signal. We would like to emphasize that present day studies on whole genome sequencing data rarely consists of heterogeneous sequencing data such as the 1000 Genomes Project. As a general quality step, we recommend to apply genomic sites filters when analyzing sequencing data to reduce false positive variant detection. A common filter is based on abnormal site depth across the samples, this filter excludes low quality and low complexity regions for the reference (see “Analyses on low coverage data” section). For sequencing data mapped to a reference of lesser quality, excluding repetitive genomic regions can further reduce the false positive SNP detection.

In conclusion, we have implemented two PC-based test statistics to perform selection scans in the PCAngsd framework that performs iterative inference of population structure based on either GL or genotype data. This makes it possible to scan for selection genome-wide in data sets of low and/or variable coverage data sampled from genetically continuous populations. We show that the signatures of selection obtained from the low coverage in both the East Asian and European populations were on par with those from the high quality genotype data obtained from existing state-of-the-art software using called genotypes. The PCAngsd framework also reduces the need to rely on ad-hoc filters on SNP sites and/or samples. All obtained candidates for selection identified from the low-coverage data have been described in other studies targeting signatures of selection in European and East Asian ancestries. The PCAngsd framework is freely available at https://github.com/rosemeis/pcangsd.

## Supplementary Information

**Additional file 1.Figure S1:** Mean depth of coverage of the low coverage data from the 1000 Genomes Project with East Asian and European ancestries used for selection scans. **Figure S2:** PCAngsd results on the high quality genotype dataset of the Asian populations in the 1000 Genomes Project. PCA plot of the four Asian populations showing the separation of Northern and Southern Asia on PC1 and PC2 separating KHV and CDX (A). QQ-plot of the test statistics, including PCAngsd-S2 statistics before and after genomic inflation correction (B). Manhattan plot of the selection scan of PC1 (C) and PC2 (D) based on the PCAngsd-S1 statistic and PCAngsd-S2 (E) of both PCs. Manhattan plots from PCAngsd-S2 has been corrected for genomic inflation. Red horizontal line is the Bonferroni adjusted significance level. **Figure S3:** PCAngsd results on the high quality genotype dataset of the European populations in the 1000 Genomes Project. PCA plot of the four European populations showing the separation of Northern and Southern Europe on PC1 (A). QQ-plot of the test statistics, including PCAngsd-S2 statistics before and after genomic inflation correction (B). Manhattan plot of the selection scan based on the PCAngsd-S1 (C) and PCAngsd-S2 (D) test statistics along PC1. Manhattan plots from PCAngsd-S2 has been corrected for genomic inflation. Red horizontal line is the Bonferroni adjusted significance level. **Figure S4:** QQ-plots and Manhattan plots of the selection statistics from FastPCA [1] and pcadapt [3] applied to the four East Asian populations obtained. Red horizontal line is the Bonferroni adjusted significance level. pcadapt has been corrected for genomic inflation. CG standard: Called genotypes from low-coverage data with a genotype quality threshold on 20. **Figure S5:** QQ-plots and Manhattan plots of the selection statistics from FastPCA and pcadapt applied to the four European populations obtained. Red horizontal line is the Bonferroni adjusted significance level. pcadapt has been corrected for genomic inflation. CG standard: Called genotypes from low-coverage data with a genotype quality threshold on 20. **Figure S6:** PCA plot, QQ-plots and Manhattan plots of the selection statistics obtained from PCAngsd, FastPCA and pcadapt applied to a European (CEU), Asian (CHB), and African (AFR) population. Red horizontal line is the Bonferroni adjusted significance level. Only one PCA plot is shown as they were all identical. pcadapt has been corrected for genomic inflation. HQG: High quality genotype data. **Figure S7:** Read length bias in the low-coverage sequencing data of the East Asian populations. (AB) PCA plots of the data only filtered using a callability filter, where in (A) individuals are colored by population, and (B) displays the individuals colored by sequencing read length. (C-D) PCA plots of the data filtered by a callability filter and corrected for read length bias. **Figure S8:** No read length bias in the low-coverage sequencing data of the European populations. (A-B) PCA plots of the data filtered using a callability filter, where in (A) individuals are colored by population, and (B) displays the individuals colored by sequencing read length. **Figure S9:** PCA plot, QQ plots and Manhattan plots of the selection statistics obtained from PCAngsd applied to the four East Asian populations for SNPs called from the low-coverage sequencing data using ANGSD [2]. The called SNPs have additionally been filtered using a callability filter and corrected for readlength bias. Red horizontal line is the Bonferroni adjusted significance level. **Figure S10:** PCA plot, QQ plots and Manhattan plots of the selection statistics obtained from PCAngsd applied to the four European populations for SNPs called from the low-coverage sequencing data using ANGSD [2]. The called SNPs have additionally been filtered using a callability filter. Red horizontal line is the Bonferroni adjusted significance level. **Figure S11:** Downsampling to 0.5 fraction of the reads of the low-coverage sequencing data. PCA plot, QQ plots and Manhattan plots of the selection statistics obtained from PCAngsd applied to the four East Asian populations for SNPs called from the downsampled low-coverage sequencing data using ANGSD [2]. Red horizontal line is the Bonferroni adjusted significance level. **Figure S12:** Downsampling to 0.5 fraction of the reads of the low-coverage sequencing data. PCA plot, QQ plots and Manhattan plots of the selection statistics obtained from PCAngsd applied to the four European populations for SNPs called from the downsampled low-coverage sequencing data using ANGSD [2]. Red horizontal line is the Bonferroni adjusted significance level. **Figure S13:** PCA plots from PCAngsd based on the low-coverage sequencing datasets with individuals colored by their estimated individual allele frequencies in the top hits for the East Asian and European populations, respectively. The individual allele frequencies reveal the direction of the PC-based selection signals in regards to the reference allele. (A) shows the top significant hit on PC1 for the East Asian populations for the LILRA3 region (rs434124), and (B) shows the top significant hit on PC1 for the European for the LCT/MCM6 region (rs6754311).

## Data Availability

The selection statistics are integrated in the PCAngsd framework which is freely available at https://github.com/Rosemeis/pcangsd. The datasets used from the phase 3 release of the 1000 Genomes Project are available at ftp://ftp.1000genomes.ebi.ac.uk/vol1/ftp/phase3/data/ and ftp://ftp.1000genomes.ebi.ac.uk/vol1/ftp/release/20130502/ for the low-coverage sequencing data and high quality genotypes, respectively. Callability filters are available at: ftp://ftp.1000genomes.ebi.ac.uk/vol1/ftp/release/20130502/supporting/accessible_genome_masks/StrictMask.

## References

[1] Bersaglieri T, Sabeti PC, Patterson N, Vanderploeg T, Schaffner SF, Drake JA, Rhodes M, Reich DE, Hirschhorn JN (2004). Genetic signatures of strong recent positive selection at the lactase gene. Am J Hum Genet.

[2] Cheng JY, Racimo F, Nielsen R. Ohana: detecting selection in multiple populations by modelling ancestral admixture components. BioRxiv, 2019;546408. 10.1093/molbev/msab294 PMC8763095 34626111

[3] Chiang CW, Mangul S, Robles C, Sankararaman S. A comprehensive map of genetic variation in the world’s largest ethnic Group-Han Chinese. Mol Biol Evol. 2018;35(11):2736–50. 10.1093/molbev/msy170 PMC6693441 30169787

[4] 1000 Genomes Project Consortium et al. A global reference for human genetic variation. Nature, 2015;526(7571):68. 10.1038/nature15393 PMC4750478 26432245

[5] Devlin B, Roeder K (1999). Genomic control for association studies. Biometrics.

[6] Fan S, Hansen MEB, Lo Y, Tishkoff SA (2016). Going global by adapting local: a review of recent human adaptation. Science.

[7] Galinsky KJ, Bhatia G, Loh PR, Georgiev S, Mukherjee S, Patterson NJ, Price AL (2016). Fast principal-component analysis reveals convergent evolution of adh1b in Europe and East Asia. Am J Hum Genet.

[8] Jørsboe E, Anders A. Efficient approaches for large scale GWAS studies with genotype uncertainty. bioRxiv, p 786384, 2020.

[9] Korneliussen TS, Albrechtsen A, Nielsen R (2014). Angsd: analysis of next generation sequencing data. BMC Bioinform.

[10] Kotsakiozi P, Richardson JB, Pichler V, Favia G, Martins AJ, Urbanelli SS, Armbruster PA, Caccone A (2017). Population genomics of the Asian tiger mosquito, aedes albopictus: insights into the recent worldwide invasion. Ecol Evol.

[11] Lehoucq RB, Sorensen DC, Yang C. ARPACK users' guide: solution of large-scale eigenvalue problems with implicitly restarted Arnoldi methods. Society for Industrial and Applied Mathematics. 1998.

[12] Li H (2011). A statistical framework for SNP calling, mutation discovery, association mapping and population genetical parameter estimation from sequencing data. Bioinformatics.

[13] Li YF, Costello JC, Holloway AK, Hahn MW. “reverse ecology” and the power of population genomics. Evolution. 2008;62(12):2984–94. 10.1111/j.1558-5646.2008.00486.x PMC2626434 18752601

[14] Liu S, Huang S, Chen F, Zhao L, Yuan Y, Francis SS, Fang L, Li Z, Lin L, Liu R, Zhang Y, Xu H, Li S, Zhou Y, Davies RW, Liu Q, Walters RG, Lin K, Ju J, Korneliussen T, Yang MA, Fu Q, Wang J, Zhou L, Krogh A, Zhang H, Wang W, Chen Z, Cai Z, Yin Y, Yang H, Mao M, Shendure J, Wang J, Albrechtsen A, Jin X, Nielsen R, Xu X. Genomic analyses from non-invasive prenatal testing reveal genetic associations, patterns of viral infections, and Chinese population history. Cell. 2018;175(2):347–59. 10.1016/j.cell.2018.08.016 30290141

[15] Luu K, Bazin E, Blum MG (2017). pcadapt: an R package to perform genome scans for selection based on principal component analysis. Mol Ecol Resour.

[16] Mahalanobis PC. On the generalized distance in statistics. National Institute of Science of India. 1936.

[17] Meisner J, Albrechtsen A. Testing for Hardy–Weinberg equilibrium in structured populations using genotype or low‐depth next generation sequencing data. Mol Ecol Res. 2019;19(5):1144–52. 10.1111/1755-0998.13019 30977299

[18] Meisner J, Albrechtsen A (2018). Inferring population structure and admixture proportions in low-depth ngs data. Genetics.

[19] Momigliano P, Florin AB, Merilä J. Biases in demographic modeling affect our understanding of recent divergence. Mol Biol Evol. 2021;38(7):2967–85. 10.1093/molbev/msab047 PMC8233503 33624816

[20] Murray KD, Janes JK, Jones A, Bothwell HM, Andrew RL, Borevitz JO (2019). Landscape drivers of genomic diversity and divergence in woodland eucalyptus. Mol Ecol.

[21] Nielsen R, Korneliussen T, Albrechtsen A, Li Y, Wang J (2012). SNP calling, genotype calling, and sample allele frequency estimation from New-Generation sequencing data. PLoS ONE.

[22] Norton HL, Kittles RA, Parra E, McKeigue P, Mao X, Cheng K, Canfield VA, Bradley DG, McEvoy B, Shriver MD (2007). Genetic evidence for the convergent evolution of light skin in Europeans and East Asians. Mol Biol Evol.

[23] Pont C, Leroy T, Seidel M, Tondelli A, Duchemin W, Armisen D, Lang D, Bustos-Korts D, Goué N, Balfourier F, Molnár-Láng M, Lage J, Kilian B, Özkan H, Waite D, Dyer S, Letellier T, Alaux M. Wheat and Barley Legacy for Breeding Improvement (WHEALBI) consortium, Joanne R, Beat K, van Eeuwijk F, Manuel S, Mayer KFX, Robbie W, Nils S, Luigi C, Georg H, Gilles C, and Jérôme S. Tracing the ancestry of modern bread wheats. Nat Genet. 51(5):905–911, 2019. 10.1038/s41588-019-0393-z 31043760

[24] Privé F, Luu K, Vilhjálmsson BJ, Blum MGB. Performing highly efficient genome scans for local adaptation with R package pcadapt version 4. Mol Biol Evol. 2020;37(7):2153–4. 10.1093/molbev/msaa053 32343802

[25] Rokhlin V, Szlam A, Tygert M. A randomized algorithm for principal component analysis. SIAM J Matrix Anal Appl. 2010;31(3):1100–24.

[26] Sallé G, Doyle SR, Cortet J, Cabaret J, Berriman M, Holroyd N, Cotton JA (2019). The global diversity of haemonchus contortus is shaped by human intervention and climate. Nat Commun.

[27] Sinclair-Waters M, Bradbury IR, Morris CJ, Lien S, Kent MP, Bentzen P (2018). Ancient chromosomal rearrangement associated with local adaptation of a postglacially colonized population of Atlantic cod in the northwest Atlantic. Mol Ecol.

[28] Voight BF, Kudaravalli S, Wen X, Pritchard JK (2006). A map of recent positive selection in the human genome. PLoS Biol.

[29] Wang H, Vieira FG, Crawford JE, Chu C, Nielsen R (2017). Asian wild rice is a hybrid swarm with extensive gene flow and feralization from domesticated rice. Genome Res.

[30] Wilder AP, Palumbi SR, Conover DO, Therkildsen NO (2020). Footprints of local adaptation span hundreds of linked genes in the Atlantic silverside genome. Evol Lett.

[31] Yi X, Liang Y, Huerta-Sanchez E, Jin X, Cuo ZX, Pool JE, Xu X, Jiang H, Vinckenbosch N, Korneliussen TS, Zheng H, Liu T, He W, Li K, Luo R, Nie X, Wu H, Zhao M, Cao H, Zou J, Shan Y, Li S, Qi Y, Asan, NP, Tian G, Xu J, Liu X, Jiang T, Wu R, Zhou G, Tang M, Qin J, Wang T, Feng S, Li, H, Jiangbai L, Wang W, Chen F, Wang Y, Zheng X, Li Z, Bianba Z, Yang G, Wang X, Tang S, Gao G, Chen Y, Luo Z, Gusang L, Cao Z, Zhang Q, Ouyang W, Ren X, Liang H, Zheng H, Huang Y, Li J, Bolund L, Kristiansen K, Li Y, Zhang Y, Zhang X, Li R, Li S, Yang H, Nielsen R, Wang J, Wang J. Sequencing of 50 human exomes reveals adaptation to high altitude. Science, 2010;329(5987):75–78. 10.1126/science.1190371 PMC3711608 20595611

