# Supplementary material for: Detecting selection in low-coverage high-throughput sequencing data using principal component analysis
Source: BMC Bioinformatics. 2021 Sep 29;22:470. doi: 10.1186/s12859-021-04375-2 (PMC8480091; doi:10.1186/s12859-021-04375-2)
Supplement: Supplementary file 1 — Additional file 1.Figure S1: Mean depth of coverage of the low coverage data from the 1000 Genomes Project with East Asian and European ancestries used for selection scans. Figure S2: PCAngsd results on the high quality genotype dataset of the Asian populations in the 1000 Genomes Project. PCA plot of the four Asian populations showing the separation of Northern and Southern Asia on PC1 and PC2 separating KHV and CDX (A). QQ-plot of the test statistics, including PCAngsd-S2 statistics before and after genomic inflation correction (B). Manhattan plot of the selection scan of PC1 (C) and PC2 (D) based on the PCAngsd-S1 statistic and PCAngsd-S2 (E) of both PCs. Manhattan plots from PCAngsd-S2 has been corrected for genomic inflation. Red horizontal line is the Bonferroni adjusted significance level. Figure S3: PCAngsd results on the high quality genotype dataset of the European populations in the 1000 Genomes Project. PCA plot of the four European populations showing the separation of Northern and Southern Europe on PC1 (A). QQ-plot of the test statistics, including PCAngsd-S2 statistics before and after genomic inflation correction (B). Manhattan plot of the selection scan based on the PCAngsd-S1 (C) and PCAngsd-S2 (D) test statistics along PC1. Manhattan plots from PCAngsd-S2 has been corrected for genomic inflation. Red horizontal line is the Bonferroni adjusted significance level. Figure S4: QQ-plots and Manhattan plots of the selection statistics from FastPCA [1] and pcadapt [3] applied to the four East Asian populations obtained. Red horizontal line is the Bonferroni adjusted significance level. pcadapt has been corrected for genomic inflation. CG standard: Called genotypes from low-coverage data with a genotype quality threshold on 20. Figure S5: QQ-plots and Manhattan plots of the selection statistics from FastPCA and pcadapt applied to the four European populations obtained. Red horizontal line is the Bonferroni adjusted significance level. pcadapt has been co [file 12859_2021_4375_MOESM1_ESM.pdf]

Supplementary Material

Detecting Selection in Low-Coverage High-Throughput Sequencing Data using Principal Component Analysis

Jonas Meisner, Anders Albrechtsen, Kristian Hanghøj

The Bioinformatics Centre, Department of Biology, University of Copenhagen, Denmark

Posterior expectation of the genotype

We are using the iterative algorithm in `PCAngsd` to estimate individual allele frequencies π [4]. With the assumption of Hardy-Weinberg proportions, we can derive the posterior genotype probability using the genotype likelihoods as follows for individual i in site j:

$$P(G_{ij} = g \mid X_{ij}, \hat{\pi}_{ij}) = \frac{P(X_{ij} \mid G_{ij} = g) P(G_{ij} = g \mid \hat{\pi}_{ij})}{\sum_{g'=0}^{2} P(X_{ij} \mid G_{ij} = g') P(G_{ij} = g' \mid \hat{\pi}_{ij})}, \tag{S1}$$

$$P(G_{ij} = g \mid \hat{\pi}_{ij}) = \begin{cases} \hat{\pi}_{ij}^2, \quad g = 0, \\ 2\hat{\pi}_{ij}(1 - \hat{\pi}_{ij}), \quad g = 1, \\ (1 - \hat{\pi}_{ij})^2, \quad g = 2. \end{cases}$$

Here g is the genotype and $P(X \mid G = g)$ is the genotype likelihood. The posterior expectation of the genotype is thus given by:

$$\mathbb{E}[G_{ij} \mid X_{ij}, \hat{\pi}_{ij}] = \sum_{g=0}^{2} g \, P(G_{ij} = g \mid X_{ij}, \hat{\pi}_{ij}), \tag{S2}$$

which we use in our selection statistics to account for uncertainty in the genotypes in low-coverage data.

Supplementary figures

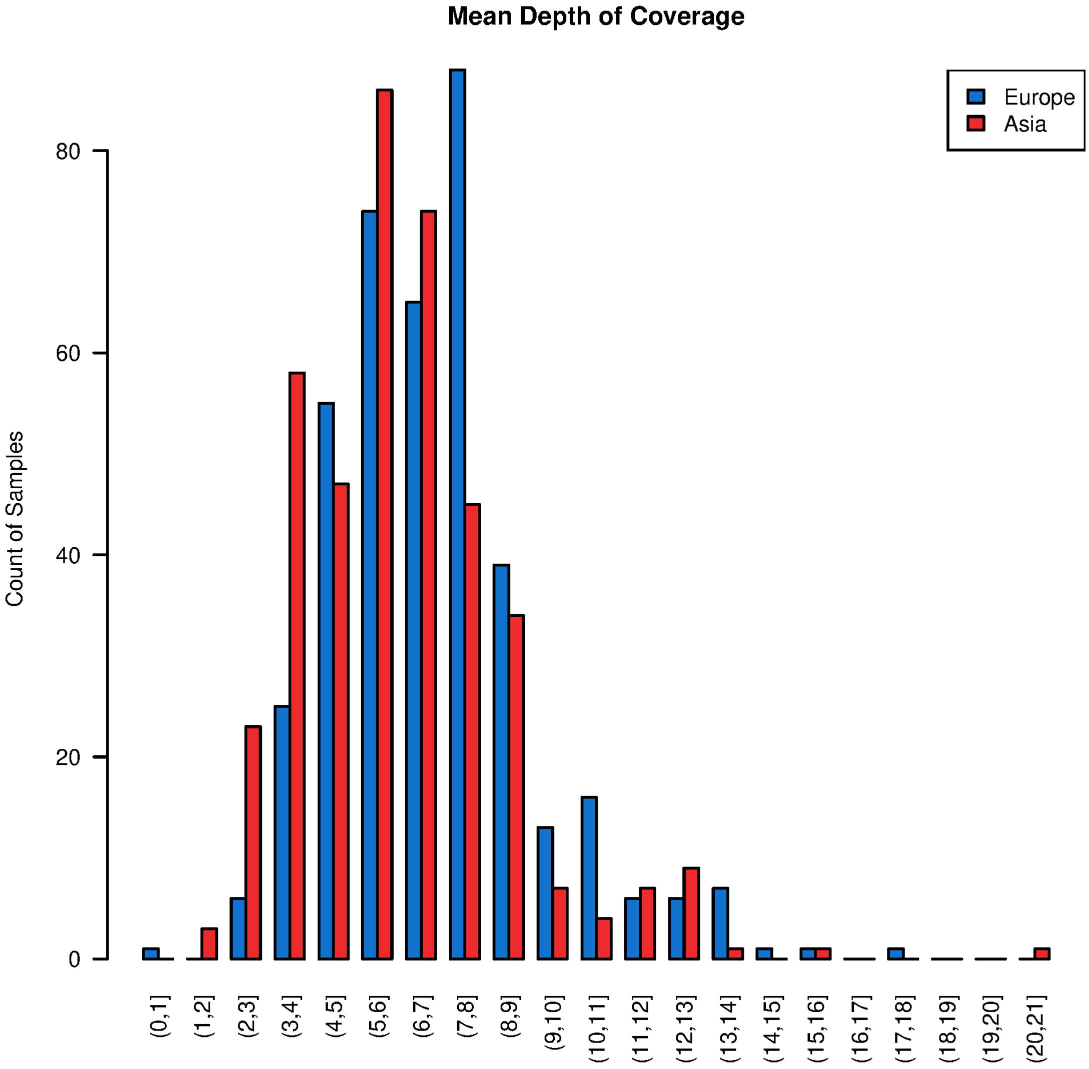

Figure S1: Mean depth of coverage of the low coverage data from the 1000 Genomes Project with East Asian and European ancestries used for selection scans.

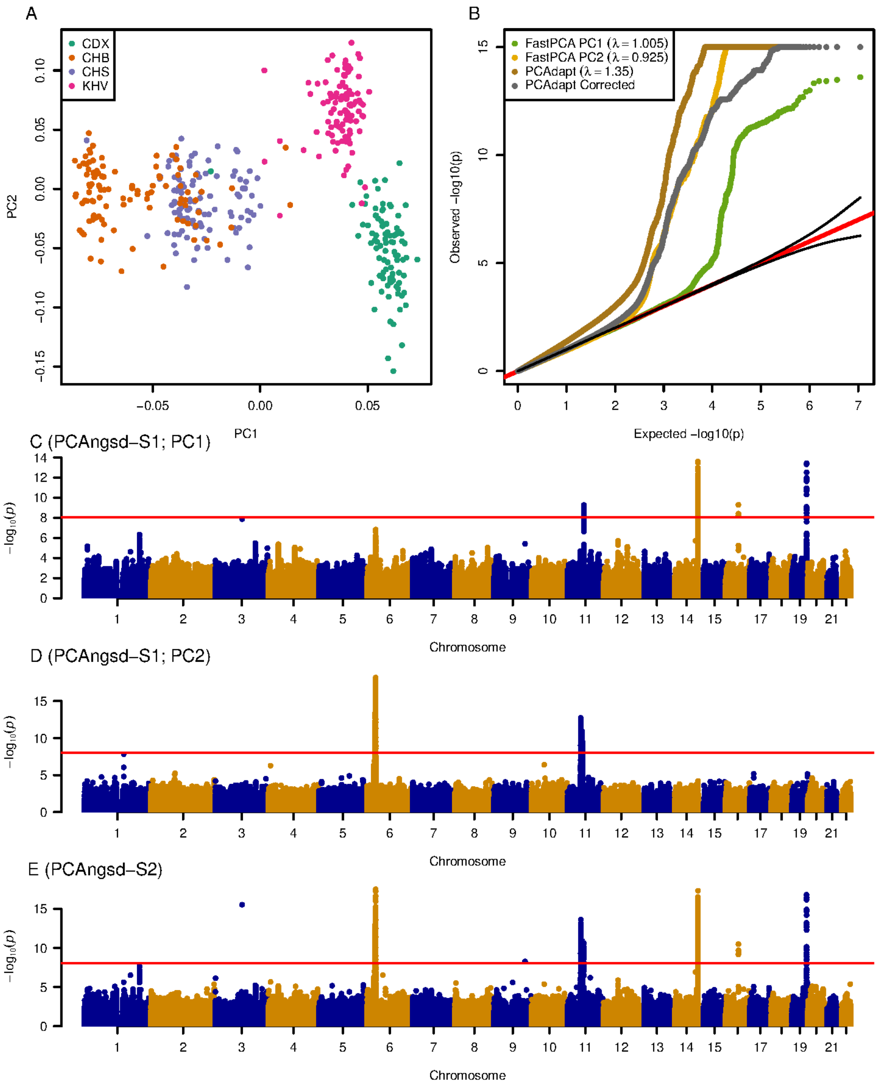

Figure S2: `PCAngsd` results on the high quality genotype dataset of the Asian populations in the 1000 Genomes Project. PCA plot of the four Asian populations showing the separation of Northern and Southern Asia on PC1 and PC2 separating KHV and CDX *(A)*. QQ-plot of the test statistics, including `PCAngsd-S2` statistics before and after genomic inflation correction *(B)*. Manhattan plot of the selection scan of PC1 *(C)* and PC2 *(D)* based on the `PCAngsd-S1` statistic and `PCAngsd-S2` *(E)* of both PCs. Manhattan plots from `PCAngsd-S2` has been corrected for genomic inflation. Red horizontal line is the Bonferroni adjusted significance level.

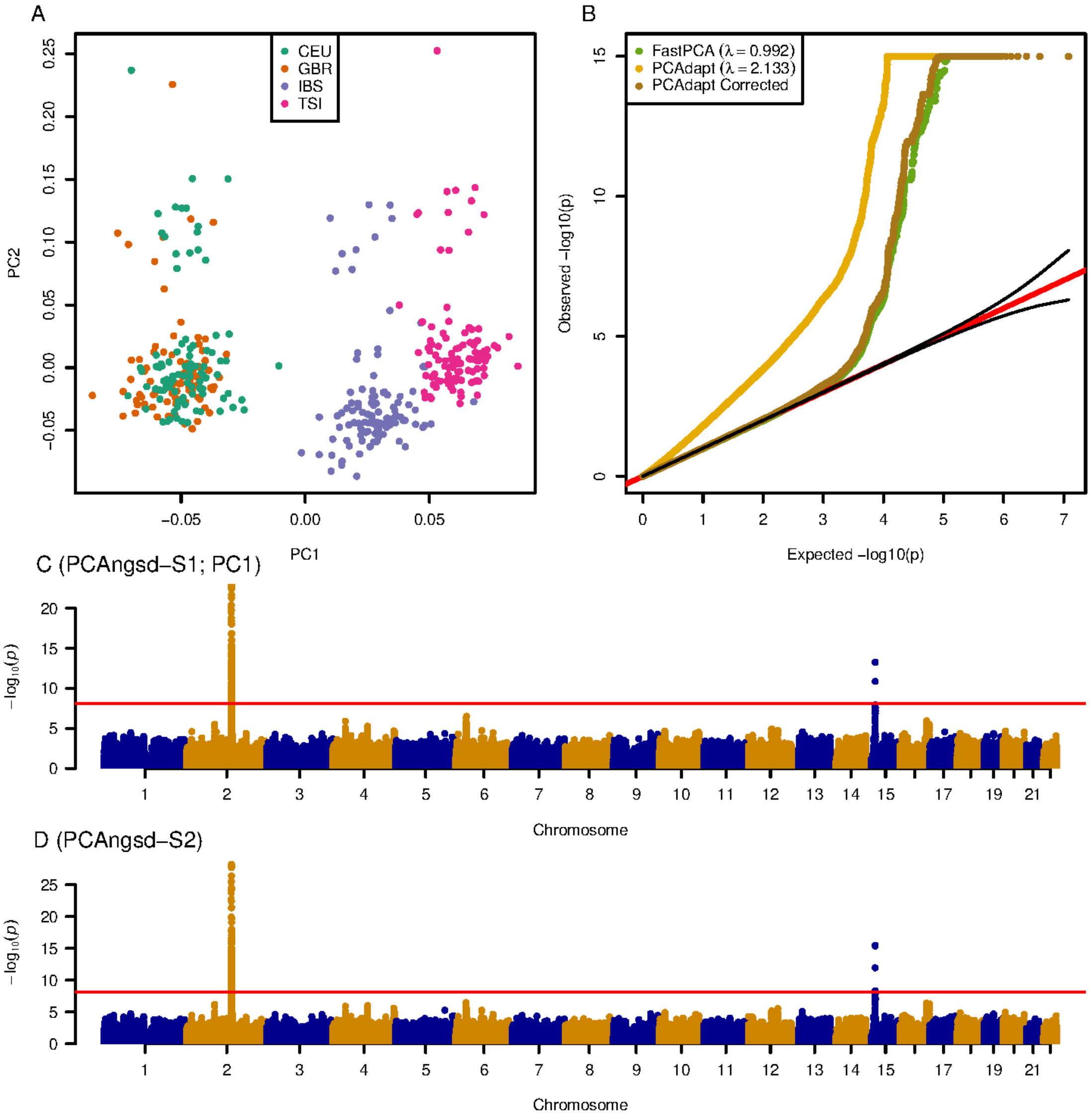

Figure S3: `PCAngsd` results on the high quality genotype dataset of the European populations in the 1000 Genomes Project. PCA plot of the four European populations showing the separation of Northern and Southern Europe on PC1 *(A)*. QQ-plot of the test statistics, including `PCAngsd-S2` statistics before and after genomic inflation correction *(B)*. Manhattan plot of the selection scan based on the `PCAngsd-S1` *(C)* and `PCAngsd-S2` *(D)* test statistics along PC1. Manhattan plots from `PCAngsd-S2` has been corrected for genomic inflation. Red horizontal line is the Bonferroni adjusted significance level.

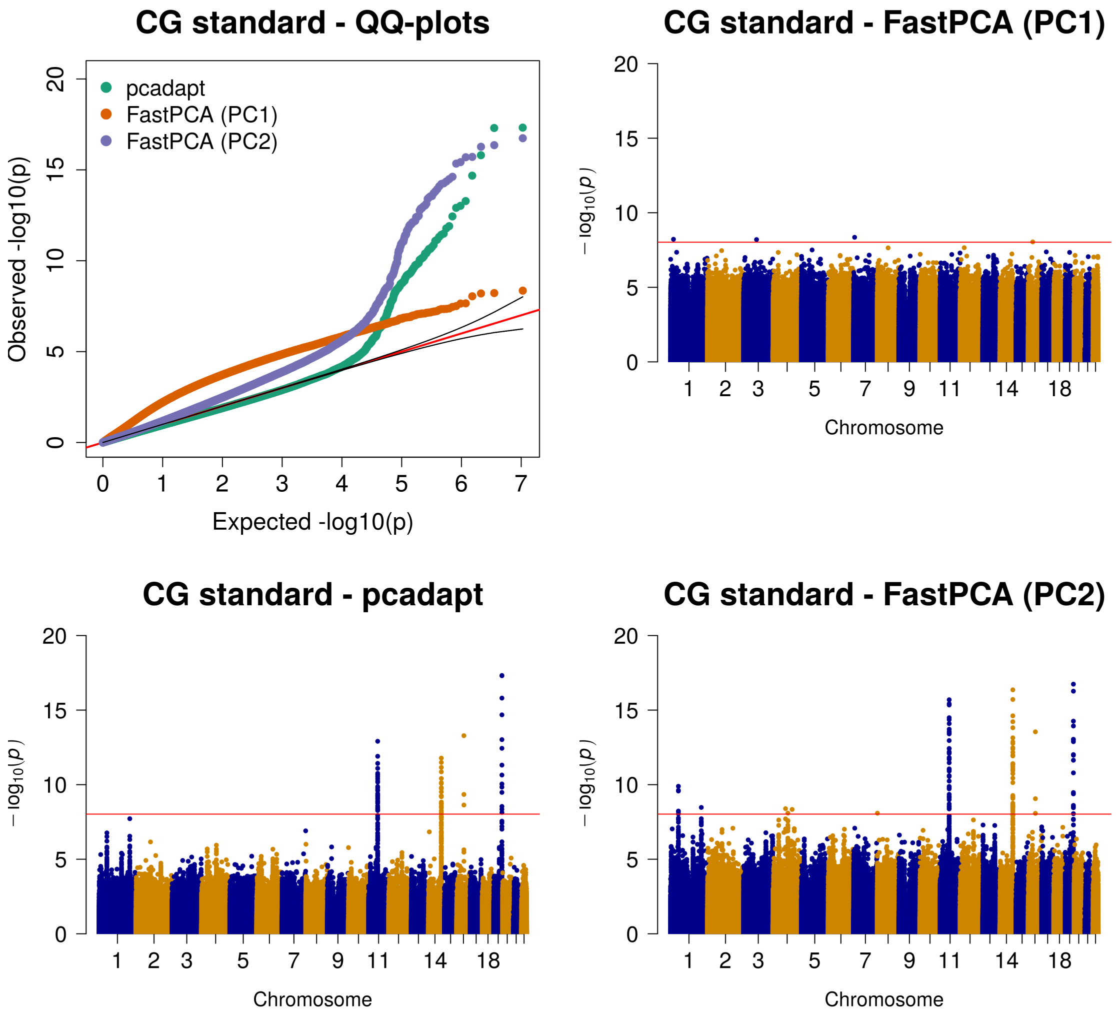

Figure S4: QQ-plots and Manhattan plots of the selection statistics from `FastPCA` [1] and `pcadapt` [3] applied to the four East Asian populations obtained. Red horizontal line is the Bonferroni adjusted significance level. `pcadapt` has been corrected for genomic inflation. `CG standard`: Called genotypes from low-coverage data with a genotype quality threshold on 20.

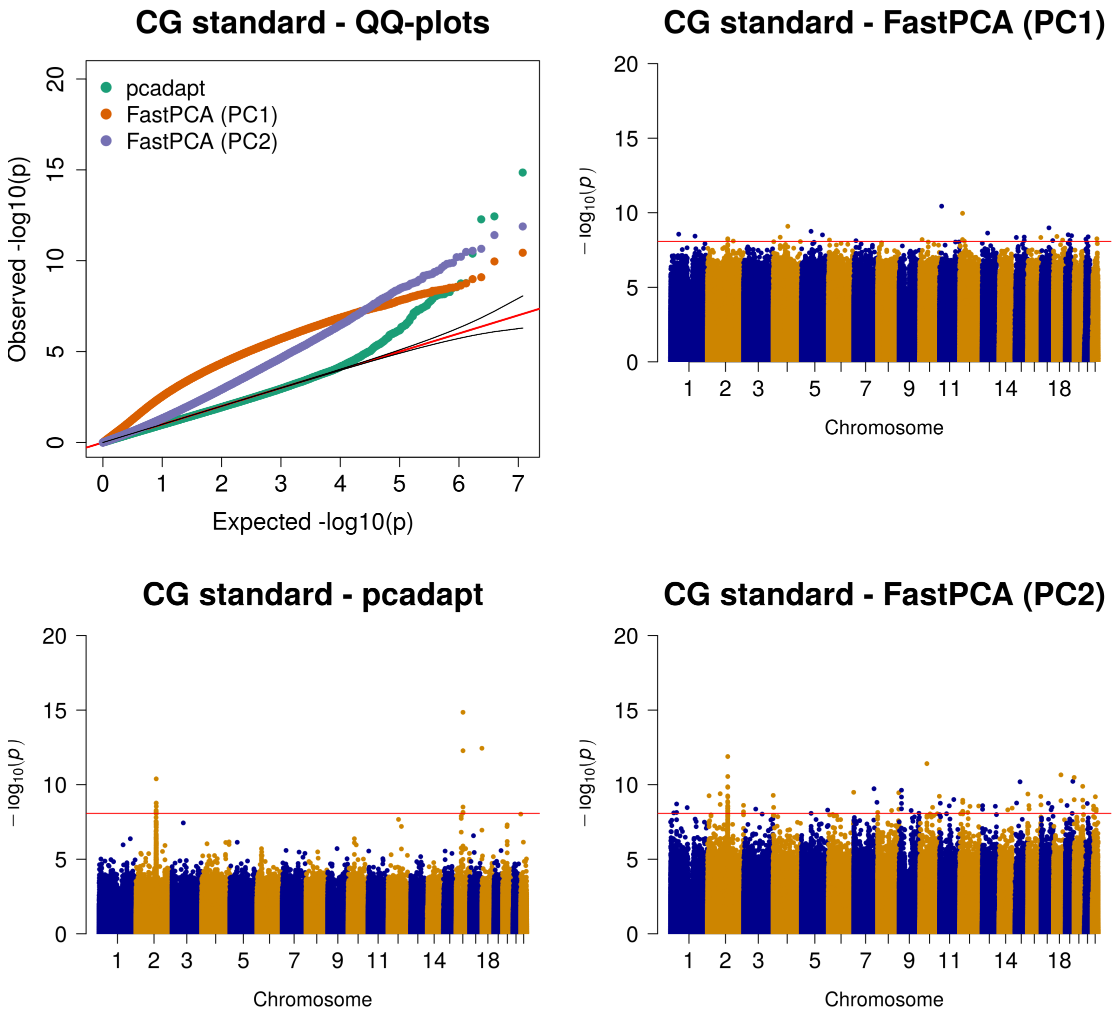

Figure S5: QQ-plots and Manhattan plots of the selection statistics from `FastPCA` and `pcadapt` applied to the four European populations obtained. Red horizontal line is the Bonferroni adjusted significance level. `pcadapt` has been corrected for genomic inflation. `CG standard`: Called genotypes from low-coverage data with a genotype quality threshold on 20.

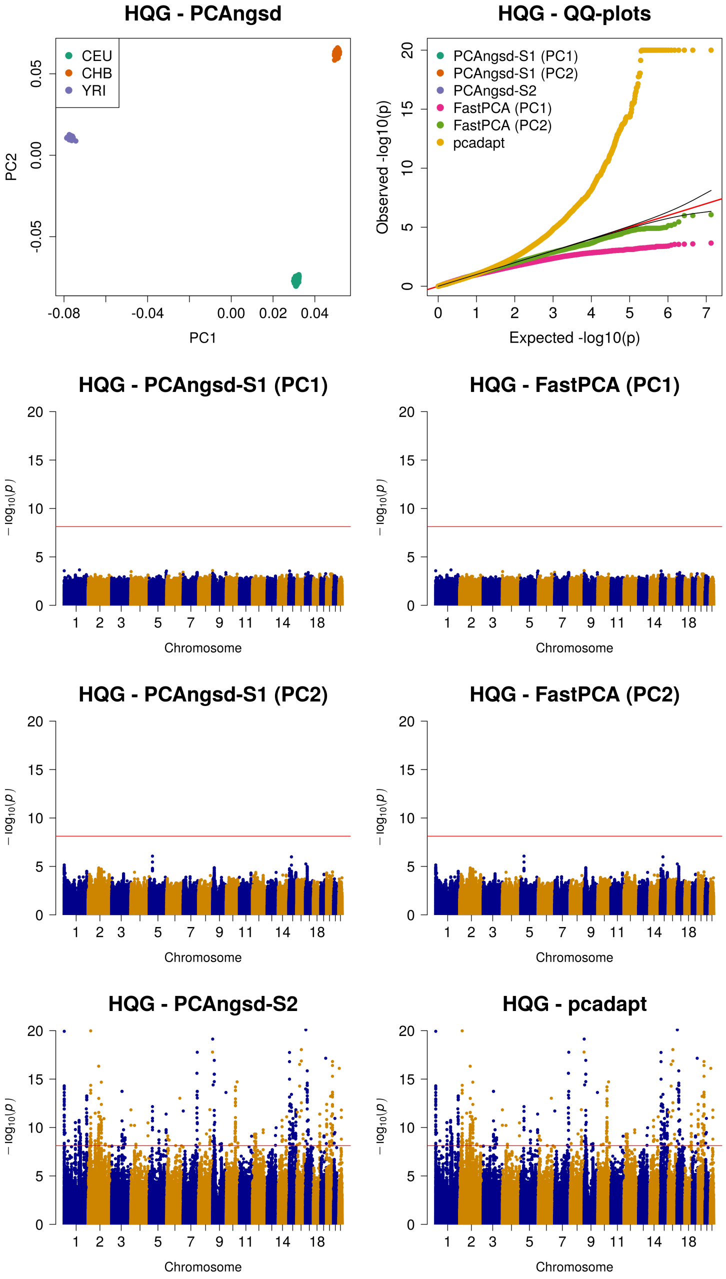

Figure S6: PCA plot, QQ-plots and Manhattan plots of the selection statistics obtained from `PCAngsd`, `FastPCA` and `pcadapt` applied to a European (CEU), Asian (CHB), and African (AFR) population. Red horizontal line is the Bonferroni adjusted significance level. Only one PCA plot is shown as they were all identical. `pcadapt` has been corrected for genomic inflation. `HQG`: High quality genotype data.

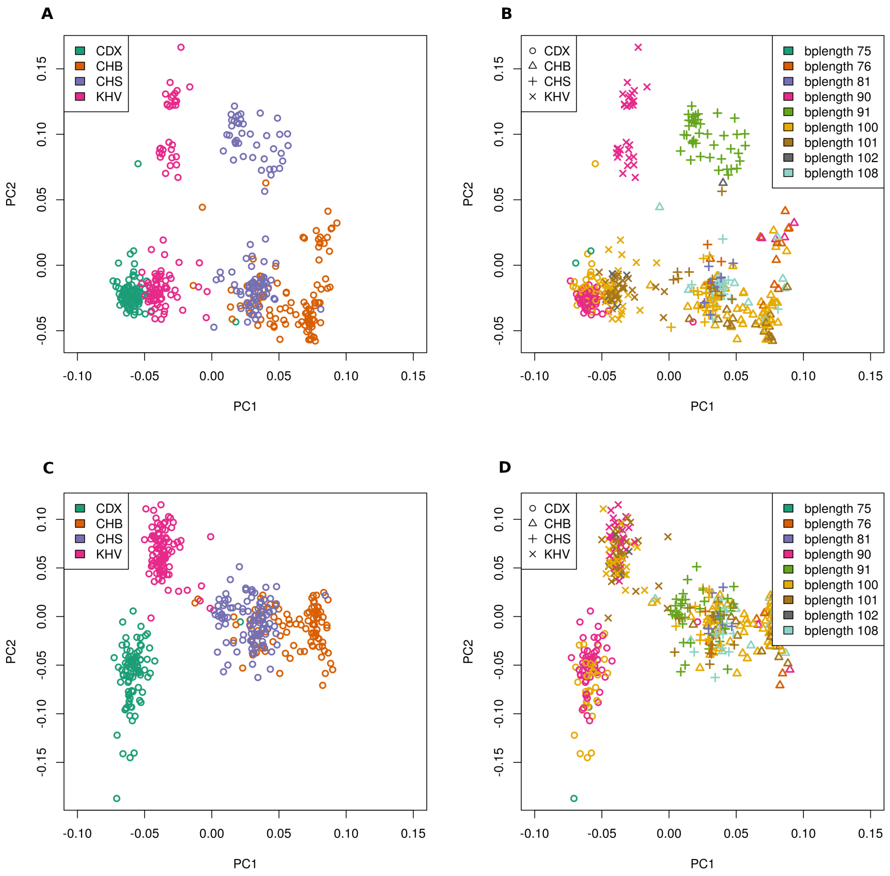

Figure S7: Read length bias in the low-coverage sequencing data of the East Asian populations. *(A-B)* PCA plots of the data only filtered using a callability filter, where in *(A)* individuals are colored by population, and *(B)* displays the individuals colored by sequencing read length. *(C-D)* PCA plots of the data filtered by a callability filter and corrected for read length bias.

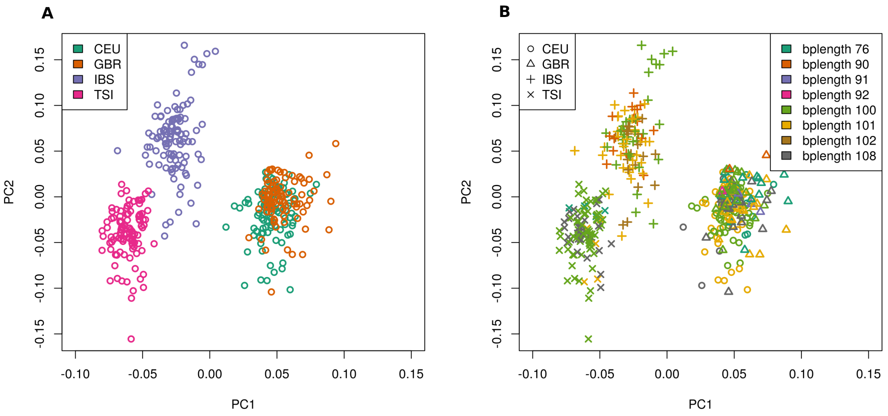

Figure S8: No read length bias in the low-coverage sequencing data of the European populations. *(A-B)* PCA plots of the data filtered using a callability filter, where in *(A)* individuals are colored by population, and *(B)* displays the individuals colored by sequencing read length.

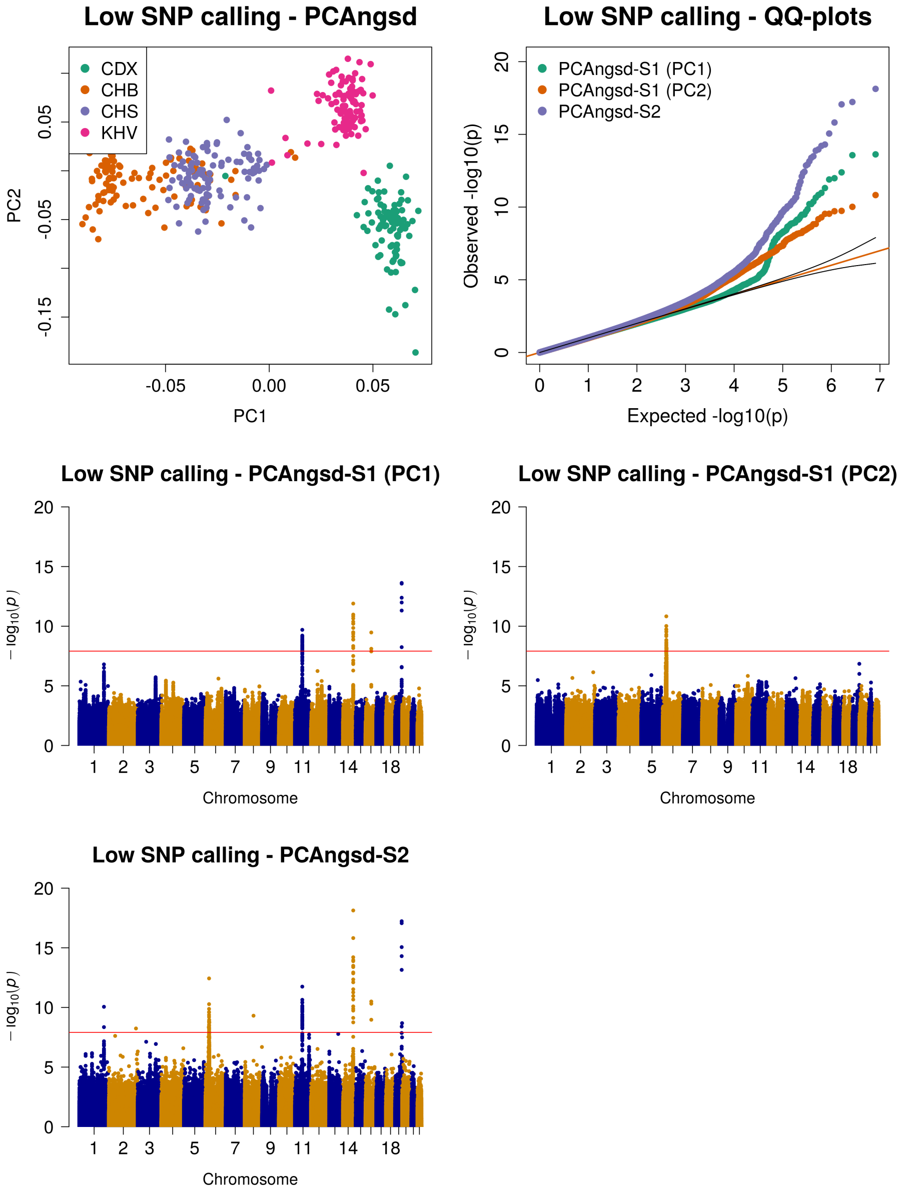

Figure S9: PCA plot, QQ plots and Manhattan plots of the selection statistics obtained from `PCAngsd` applied to the four East Asian populations for SNPs called from the low-coverage sequencing data using `ANGSD` [2]. The called SNPs have additionally been filtered using a callability filter and corrected for read length bias. Red horizontal line is the Bonferroni adjusted significance level.

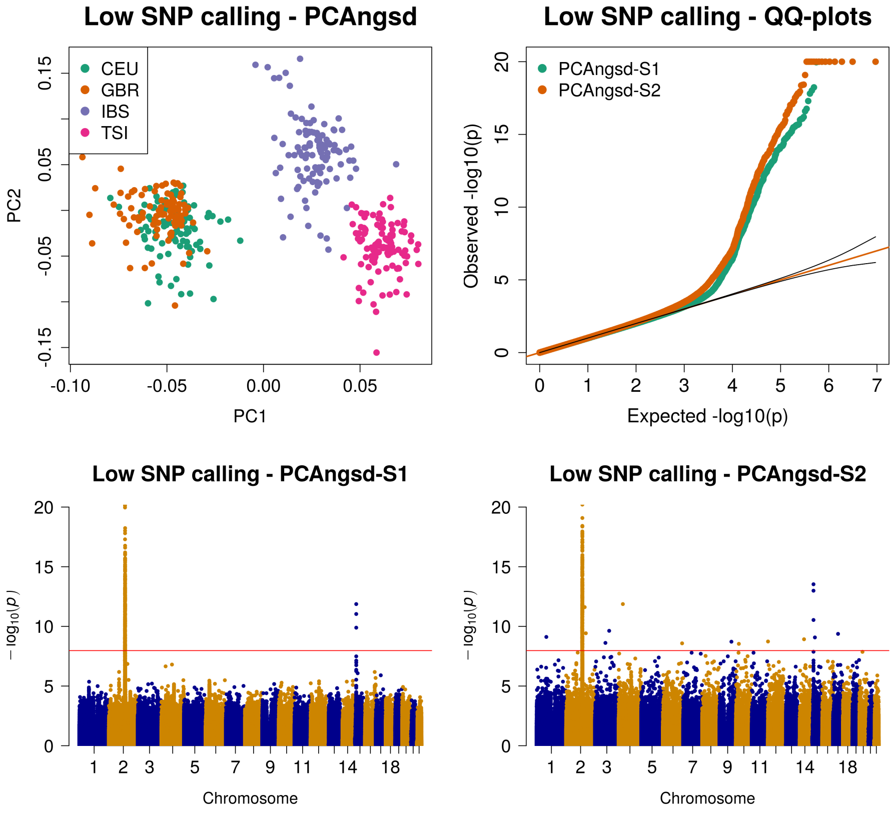

Figure S10: PCA plot, QQ plots and Manhattan plots of the selection statistics obtained from `PCAngsd` applied to the four European populations for SNPs called from the low-coverage sequencing data using `ANGSD` [2]. The called SNPs have additionally been filtered using a callability filter. Red horizontal line is the Bonferroni adjusted significance level.

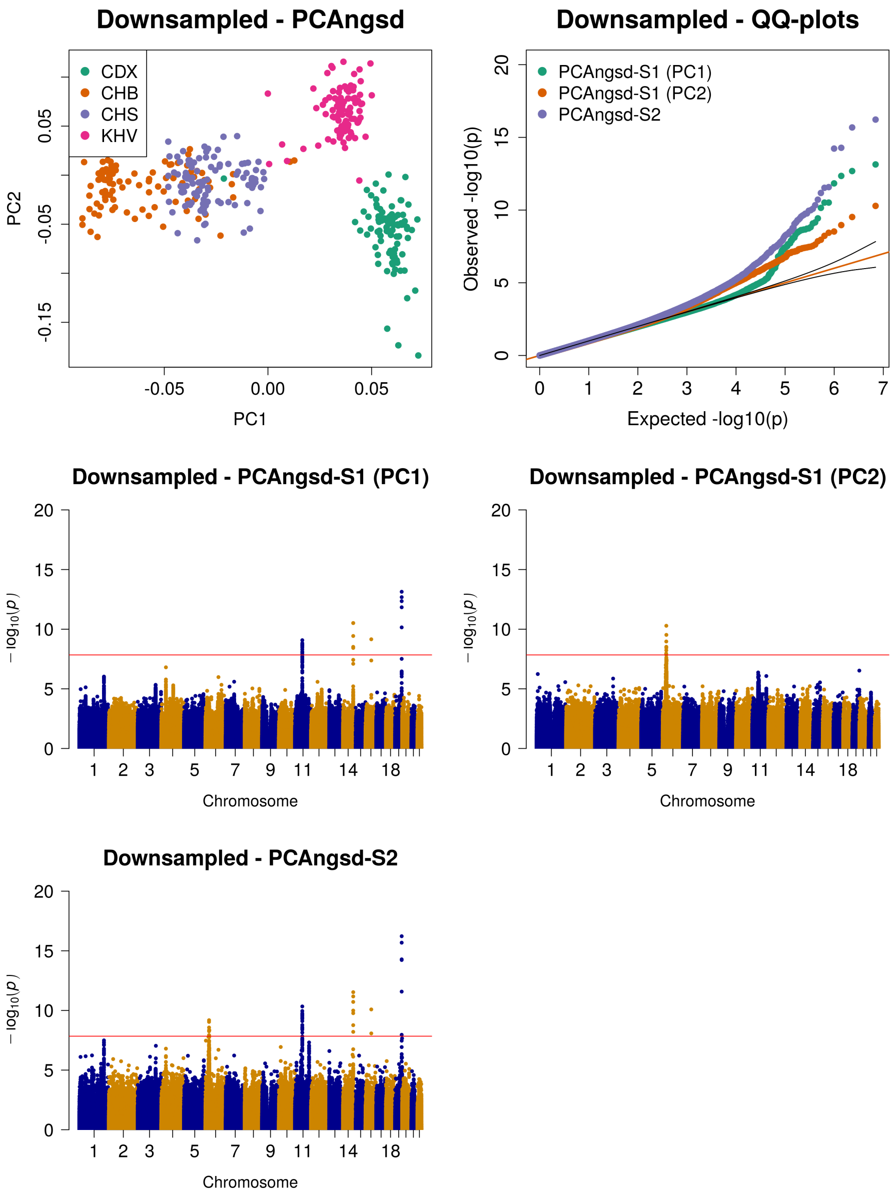

Figure S11: Downsampling to 0.5 fraction of the reads of the low-coverage sequencing data. PCA plot, QQ plots and Manhattan plots of the selection statistics obtained from `PCAngsd` applied to the four East Asian populations for SNPs called from the downsampled low-coverage sequencing data using `ANGSD` [2]. Red horizontal line is the Bonferroni adjusted significance level.

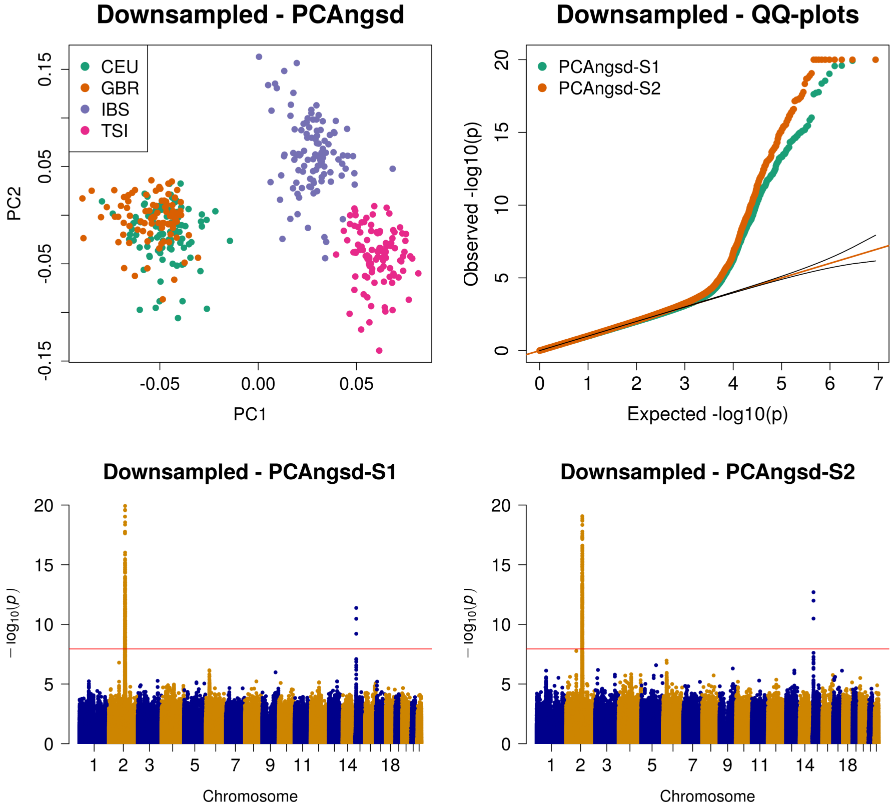

Figure S12: Downsampling to 0.5 fraction of the reads of the low-coverage sequencing data. PCA plot, QQ plots and Manhattan plots of the selection statistics obtained from `PCAngsd` applied to the four European populations for SNPs called from the downsampled low-coverage sequencing data using `ANGSD` [2]. Red horizontal line is the Bonferroni adjusted significance level.

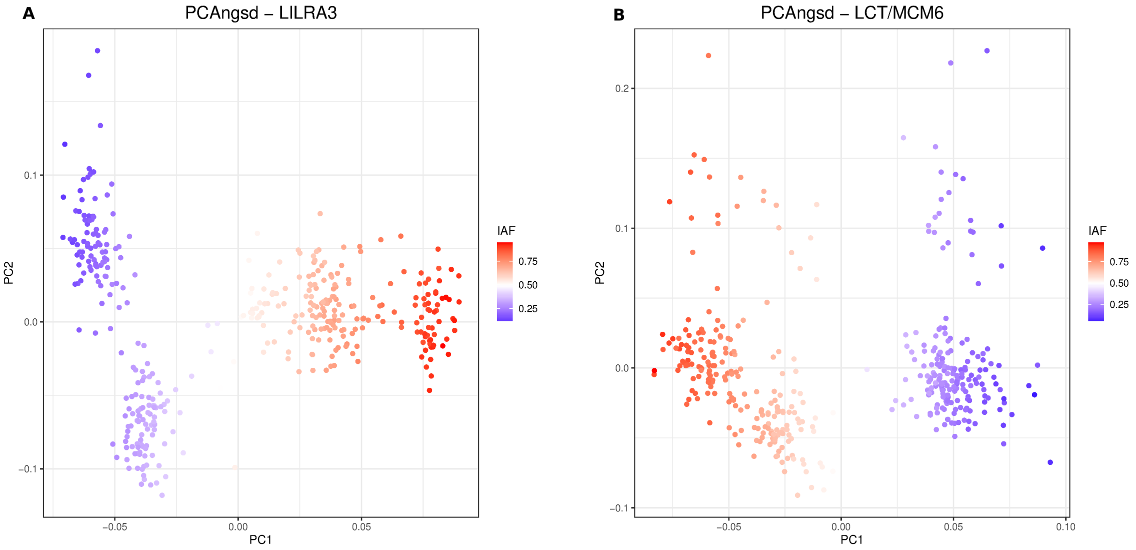

Figure S13: PCA plots from `PCAngsd` based on the low-coverage sequencing datasets with individuals colored by their estimated individual allele frequencies in the top hits for the East Asian and European populations, respectively. The individual allele frequencies reveal the direction of the PC-based selection signals in regards to the reference allele. *(A)* shows the top significant hit on PC1 for the East Asian populations for the *LILRA3* region (rs434124), and *(B)* shows the top significant hit on PC1 for the European for the *LCT/MCM6* region (rs6754311).

Chrom	ID	Position	A1	A2	F	p-value
3	rs149768401	100365528	C	G	-0.40	1.20×10^{-12}
6	rs41542812	32629931	G	C	0.086	0.42
9	rs115349067	117013044	C	A	-0.26	1.26×10^{-7}
11	rs7101761	49598178	G	A	-0.068	0.071
11	rs72643559	61620274	C	T	-0.039	0.23
14	rs1071803	106209119	T	C	0.022	1
16	rs17822931	48258198	C	T	-0.015	0.64
19	rs434124	54809336	C	G	-0.011	1

Table S1: Hardy-Weinberg equilibrium test using `PCAngsd` on the `HQG` data from the four East Asian populations. The table only contains the significant top hits from the selection analyses. F: inbreeding coefficient.

References

[1] Kevin J Galinsky, Gaurav Bhatia, Po-Ru Loh, Stoyan Georgiev, Sayan Mukherjee, Nick J Patterson, and Alkes L Price. Fast principal-component analysis reveals convergent evolution of adh1b in europe and east asia. *The American Journal of Human Genetics*, 98(3):456–472, 2016.

[2] Thorfinn Sand Korneliussen, Anders Albrechtsen, and Rasmus Nielsen. Angsd: analysis of next generation sequencing data. *BMC bioinformatics*, 15(1):356, 2014.

[3] Keurcien Luu, Eric Bazin, and Michael G B Blum. pcadapt: an R package to perform genome scans for selection based on principal component analysis. *Mol. Ecol. Resour.*, 17(1):67–77, January 2017.

[4] Jonas Meisner and Anders Albrechtsen. Inferring population structure and admixture proportions in low-depth ngs data. *Genetics*, 210(2):719–731, 2018.
